# Identification of MEDAG and SERPINE1 Related to Hypoxia in Abdominal Aortic Aneurysm Based on Weighted Gene Coexpression Network Analysis

**DOI:** 10.3389/fphys.2022.926508

**Published:** 2022-07-06

**Authors:** Biyun Teng, Chaozheng Xie, Yu Zhao, Qiu Zeng, Fangbiao Zhan, Yangyang Feng, Zhe Wang

**Affiliations:** ^1^ Department of Vascular Surgery, The First Affiliated Hospital of Chongqing Medical University, Chongqing, China; ^2^ Department of Gastrointestinal Surgery, The First Affiliated Hospital of Chongqing Medical University, Chongqing, China; ^3^ Department of Orthopedics, Chongqing University Three Gorges Hospital, Chongqing, China

**Keywords:** abdominal aortic aneurysm, hypoxia, weighted gene coexpression network analysis, gene set enrichment analysis, hub genes

## Abstract

**Purpose:** Abdominal aortic aneurysm (AAA) is a severe cardiovascular disease that often results in high mortality due to sudden rupture. This paper aims to explore potential molecular mechanisms and effective targeted therapies to prevent and delay AAA rupture.

**Methods:** We downloaded two microarray datasets (GSE98278 and GSE17901) from the Gene Expression Omnibus (GEO) database. Differential analysis and single-sample gene set enrichment analysis (ssGSEA) of hypoxia scores were performed on 48 AAA patients in GSE98278. We identified hypoxia- and ruptured AAA-related gene modules using weighted gene coexpression network analysis (WGCNA). Gene Ontology (GO) and Kyoto Encyclopedia of Genes and Genomes (KEGG) analyses were performed using the R package clusterProfiler. For candidate genes, validation was conducted on the mouse dataset GSE17901. Finally, we predicted drug candidates associated with the hub genes using the HERB Chinese medicine database.

**Results:** Eighty-two differentially expressed genes were screened in the ruptured and stable groups; 103 differentially expressed genes were identified between the high- and low-hypoxia groups; and WGCNA identified 58 differentially expressed genes. Finally, nine candidate genes were screened, including two hub genes (MEDAG and SERPINE1). We identified pathways such as cytokine–cytokine receptor interaction and T-helper 1-type immune response involved in AAA hypoxia and rupture. We predicted 93 traditional Chinese medicines (TCMs) associated with MEDAG and SERPINE1.

**Conclusion:** We identified the hypoxic molecules MEDAG and SERPINE1 associated with AAA rupture. Our study provides an additional direction for the association between hypoxia and AAA rupture.

## Introduction

Abdominal aortic aneurysm (AAA) is a common, severe cardiovascular disease that involves localized dilatation with a diameter of 3.0 cm or more and is often asymptomatic until rupture (Guirguis-Blake et al., 2019). Frequent ultrasound screening or imaging for unrelated abdominal symptoms is helpful in early diagnosis (Chaikof et al., 2018). Although the current incidence of AAA has decreased, mortality due to AAA rupture is still high at 81% (Force UPST Owens et al., 2019). At present, open surgery or endovascular aortic repair can be performed when AAA is ≥5.5 cm in diameter or expanding at a rate of 10.0 mm/year or symptoms (Chaikof et al., 2018). However, small AAAs can rupture during follow-up (Oliver-Williams et al., 2019), and there is a lack of adequate medical therapies to change the progression of AAAs (Golledge, 2019). Therefore, further exploring the pathophysiology and molecular expression differences between ruptured and unruptured AAAs is necessary to identify effective noninvasive therapeutic targets.

AAA is a multifactorial disease, and numerous studies have indicated that the occurrence and development of AAA involve immune cell inflammatory infiltration, increased oxidative stress in the aortic wall, and extracellular matrix degeneration (Emeto et al., 2016; Sakalihasan et al., 2018). Arterial wall hypoxia is an indispensable mechanism that can exacerbate neovascularization and the inflammatory response, increasing vascular vulnerability and the risk of rupture (Vijaynagar et al., 2013; Blassova et al., 2019). The hypoxic microenvironment results in the activation of hypoxia-inducible factor (HIF)-1, which can regulate the expression of several angiogenesis-related genes, including vascular endothelial growth factor (VEGF) and its receptors FLT-1, FLK-1, ANG-1, ANG-2, and TIE-2, thus affecting the state of AAA (Zimna and Kurpisz, 2015). However, the specific mechanisms of hypoxia involving AAA rupture remain unclear, and its related molecular network requires further exploration.

Weighted gene coexpression network analysis (WGCNA) is a systems biology method that identifies gene sets of interest and analyzes significant associations with phenotypes (Zhang and Horvath, 2005). WGCNA is now widely used to study many diseases, primarily in terms of cancer-like differentiation, drug treatment targets, and prognosis (Yin et al., 2020; Long et al., 2021). Currently, the hypoxia-related gene signature of AAA rupture has not been explored in detail, and an in-depth understanding of the hypoxia gene signature will help to assess the association between the degree of hypoxia and AAA rupture.

In our study, we performed bioinformatics analysis of Gene Expression Omnibus (GEO) datasets on AAA rupture, investigated the correlation between hypoxia and rupture, identified hypoxia-related molecules involved in AAA rupture, and provided new molecular targets for the assessment and treatment of AAA.

## Methods

### Data Collection

The publicly available datasets related to ruptured AAA, GSE98278 and GSE17901, were obtained from the GEO database (https://www.ncbi.nlm.nih.gov/geo/). The human dataset GSE98278 consists of 31 elective stable AAA (eAAA) and 17 ruptured AAA (rAAA) samples (Gäbel et al., 2017). The mouse dataset GSE17901 was used to validate the hub genes, which included three ruptured AAAs and four stable AAAs (Spin et al., 2011).

### Gene Set Enrichment Analysis

We used java Gene Set Enrichment Analysis (GSEA) Desktop program v3.0 to explore the functional variations of genes associated with AAA rupture (Mootha et al., 2003; Subramanian et al., 2005). The H. all.v7.4. symbols.gmt gene sets from the Molecular Signatures Database (MSigDB) (http://www.broadinstitute.org/gsea/msigdb/) were regarded as the reference gene sets, and gene set permutation was performed 1,000 times for each analysis (Subramanian et al., 2005). Values of *p* < 0.05 and false discovery rate (FDR) q < 0.25 were considered statistically significant.

### Assessment of Hypoxia Status

Two hundred genes with hypoxia signatures were acquired from MSigDB (https://www.gsea-msigdb.org/gsea/msigdb/), and the hypoxia status of AAA was assessed by the single-sample gene set enrichment analysis (ssGSEA) algorithm in the R package GSVA for the GSE98278 dataset. The patients were divided into high and low hypoxia score groups based on the median hypoxia score. The R code used for analysis is provided as Supplementary Material.

### Identification of Differentially Expressed Genes

The R package limma (version 3.3.3) was used to identify differentially expressed genes (DEGs) between eAAA and rAAA and between the high and low hypoxia score groups. |Fold change| ≥2 and *p* < 0.05 were considered significant.

### Functional Enrichment Analysis

The DEGs were subjected to enrichment analysis with Gene Ontology (GO) and Kyoto Encyclopedia of Genes and Genomes (KEGG) terms using the R package clusterProfiler (version 3.14.3) to obtain gene set enrichment results. In the GSEA runs, the minimum gene set size was set to 5, the maximum gene set size was set to 5,000, and a *p value* of <0.05 and FDR of <0.25 were considered statistically significant. To perform GO and KEGG pathway functional enrichment analyses of key module genes, we used Cytoscape (version 3.9.0, National Resource for Network Biology) with the plug-in ClueGO (version 2.5.8) (Bindea et al., 2009). In this study, functional analysis was selected as the analysis mode, *Homo sapiens* as the organism, and groups as the visual style.

### Construction of a Weighted Gene Coexpression Network and Candidate Gene Identification

First, we excluded the top 50% of genes with the smallest median absolute deviation (MAD) in the gene expression profiles. We removed outlier genes and samples using the goodSamplesGenes method of the R package WGCNA. We chose soft-threshold β = 8 (scale-free R2 = 0.89) to construct a scale-free coexpression network. Next, signature gene module identification was performed with hierarchical clustering (minimum module size was at least 30 genes). In addition, we merged modules with distances less than 0.25, resulting in 25 coexpression modules. Notably, the gray module was considered the set of genes that could not be assigned to any module.

Finally, we calculated the correlation of clinical characteristics and gene expression to obtain the gene significance (GS) and module membership (MM) by calculating the feature vector of each module and the expression correlation of the gene. Based on the cutoff criteria (|MM| > 0.7 and |GS| > 0.2), 58 genes with high connectivity in the module of clinical characteristics were identified as the key genes of the pivotal modules. Then, the intersection between DEGs and key genes was taken, and nine candidate genes were identified.

### Validation of Candidate Genes and Selection of Key Genes

The mouse gene expression profile GSE17901 was extracted to compare the expression of candidate genes in rAAA and eAAA, and genes with *p* < 0.05 were identified as hub genes. The common genes in the human dataset and mouse dataset were defined as the final hub genes.

### Construction of the Competitive Endogenous RNA Network

We retrieved miRNAs predicted by seven algorithms (TargetScan, miRanda, Pictar2, PITA, microT, miRmap, and RNA22) from the starBase database (v2.0; Sun Yat-sen University, Guangzhou, China) (Mirfakhraee et al., 1985) that may bind to the final hub genes, and the miRNAs identified by ≥ 3 of these algorithms were selected. Then, we obtained the miRNA-lncRNA target data from CLIP data (high stringency, ≥3) and performed correlation analysis with the final hub genes in gene expression profiling interactive analysis (GEPIA). We selected lncRNAs that were positively correlated with the hub genes. LncRNA–miRNA–mRNA interactions were imported into Cytoscape (version 3.9.0, National Resource for Network Biology) for ceRNA network construction.

### Prediction of Traditional Chinese Medicine

HERB (http://herb.ac.cn/) is a high-throughput experiment- and reference-guided database of TCM (Fang et al., 2021). Fisher’s exact test was used in the HERB database to infer the correlation between target genes and TCM. We imported the final key genes into HERB to explore relevant TCMs.

## Results

### Identification and Functional Enrichment Analysis of Differential Expressed Genes in Ruptured Abdominal Aortic Aneurysm and Elective Stable Abdominal Aortic Aneurysm

**FIGURE 1:**
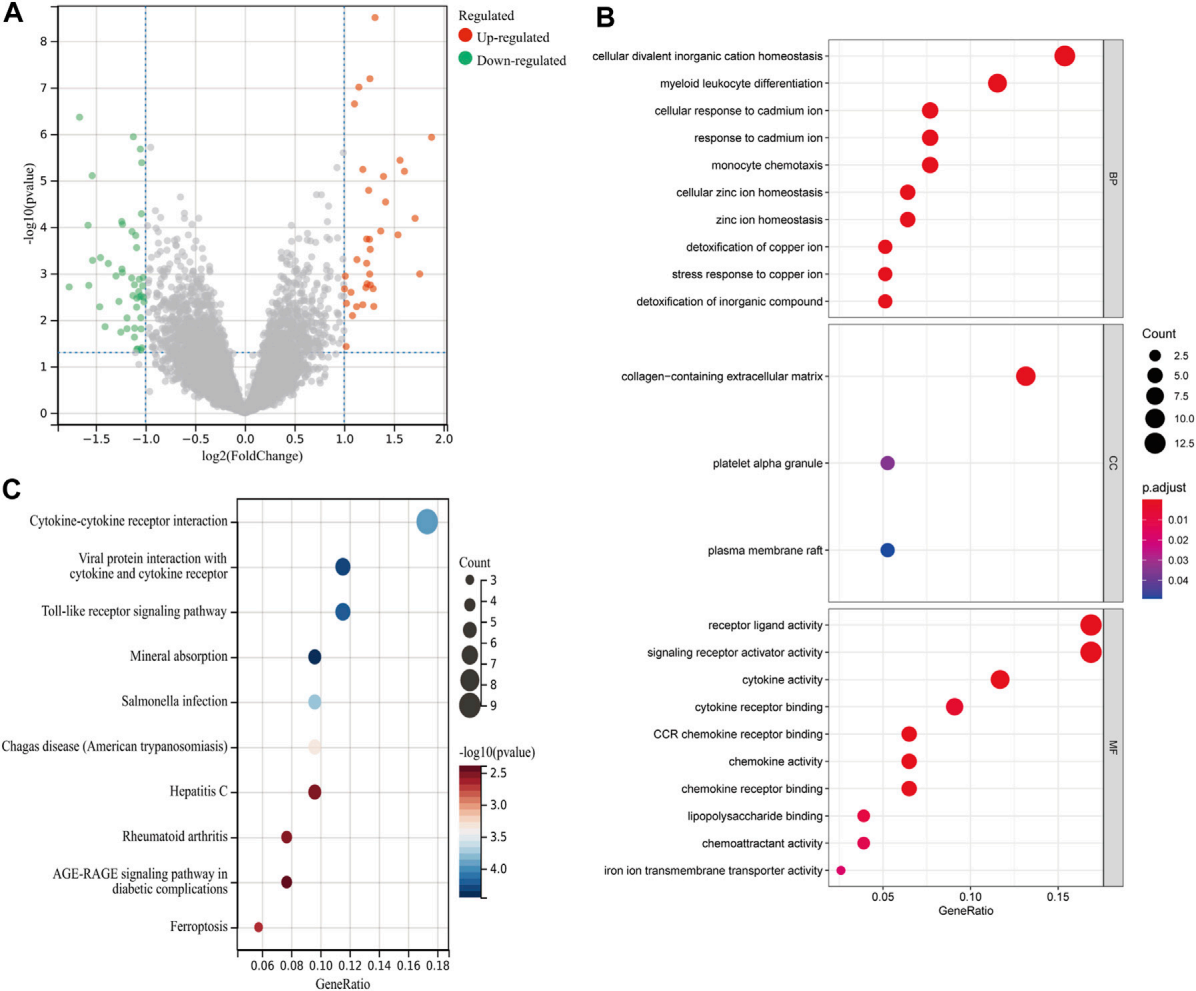
Differential analysis between rAAA and eAAA. **(A)** The scatter plot shows the DEGs between the rAAA and eAAA groups. Red represents relatively upregulated genes, and green represents downregulated genes. **(B)** Bubble plot of GO enrichment analysis of DEGs. The size of the dots corresponds to the number of genes, and the color of the dots represents the *p value*. **(C)** Bubble plots of the top 10 KEGG pathways for molecular function.

A total of 82 DEGs were identified between 17 rAAA and 31 eAAA samples, including 35 upregulated genes and 47 downregulated genes (Figure 1A). We used GO and KEGG functional enrichment analyses to explore the potential biological functions of the above DEGs. Figure 1B shows that the DEGs were mainly involved in cellular divalent inorganic cation homeostasis, myeloid leukocyte differentiation, and cellular response to cadmium ion in the biological process (BP) category; the collagen-containing extracellular matrix, platelet alpha granule, and plasma membrane raft in the cellular component (CC) category; and receptor-ligand activity, signaling receptor activator activity, and cytokine activity in the molecular function (MF) category. The KEGG enrichment analysis revealed that the DEGs were mostly enriched in cytokine–cytokine receptor interaction, viral protein interaction with cytokine and cytokine receptor, and the Toll-like receptor signaling pathway (Figure 1C).

### Identification and Functional Enrichment Analysis of Differential Expressed Genes Associated With the Hypoxic State

**FIGURE 2:**
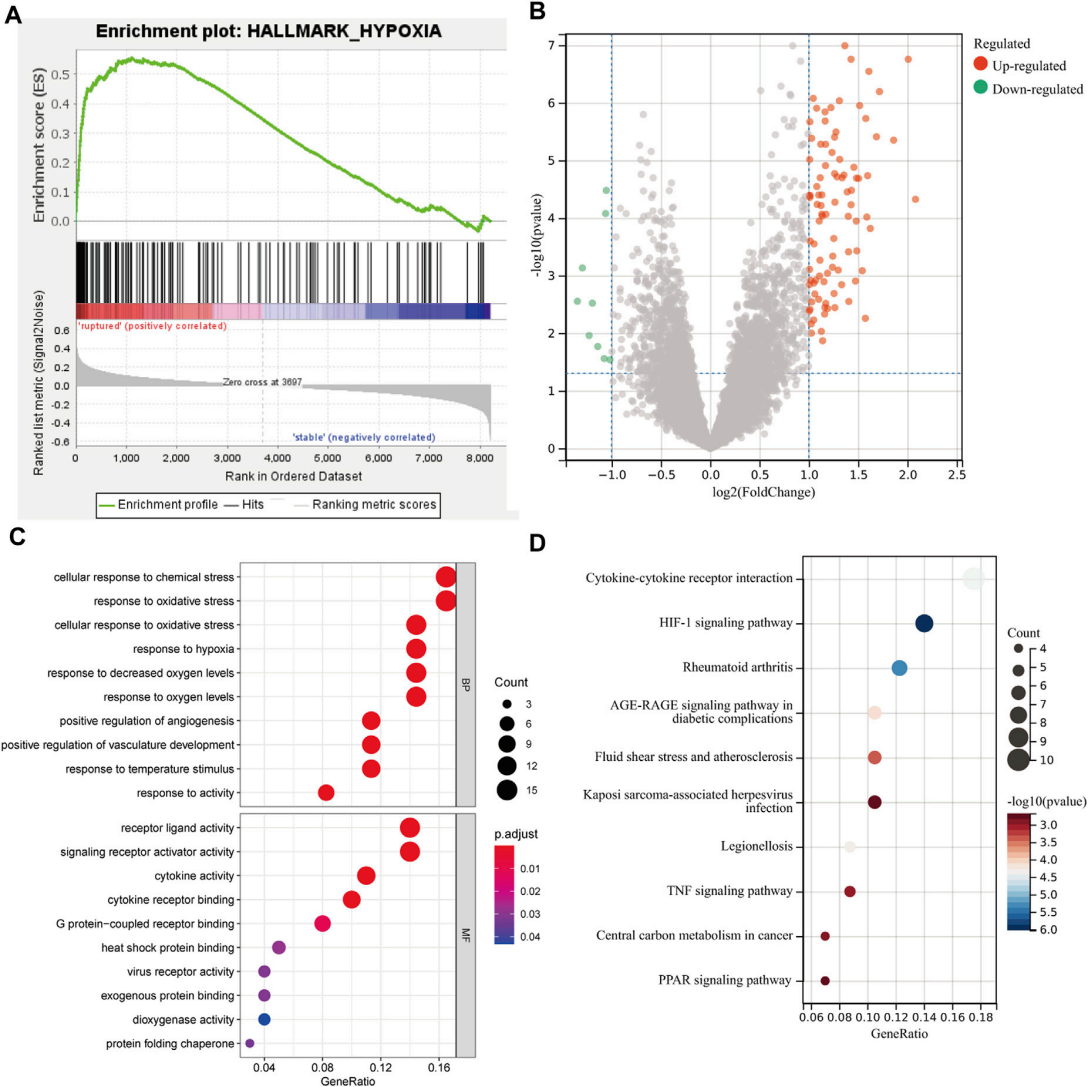
Differential and functional enrichment analyses of hypoxic states in AAA. **(A)** GSEA showed a high enrichment of hypoxic gene sets in the rupture group. **(B)** The scatter plot shows the DEGs between the high and low hypoxia scoring groups. **(C)** Bubble plot of the top 10 GO pathways by biological process and molecular function. **(D)** Bubble plot of the top 10 KEGG pathways enrichment analysis for molecular function.

The GSEA results showed that hypoxia-related genes were highly enriched in rAAA (Figure 2A), suggesting that hypoxia is involved in the process and rupture of AAA. Then, we performed hypoxia scoring in patients in the GSE98278 dataset by ssGSEA, and 48 patients were divided into two groups of high and low hypoxia. A total of 103 hypoxia-related DEGs were identified by comparing gene expression in the high- and low-hypoxia groups (Figure 2B). The hypoxia-related DEGs were subjected to GO and KEGG functional enrichment analyses, and the top 10 gene pathways are shown in Figures 2C,D. We detected enrichment in several BPs, mainly in cellular response to chemical stress, oxidative stress, and cellular response to oxidative stress; the MF analysis emphasized the role of receptor–ligand activity, signaling receptor activator activity, and cytokine activity in the DEGs. KEGG functional enrichment analysis showed that cytokine–cytokine receptor interaction, the HIF-1 signaling pathway, and rheumatoid arthritis were the most critical pathways.

### Construction of a Weighted Gene Coexpression Network

**FIGURE 3:**
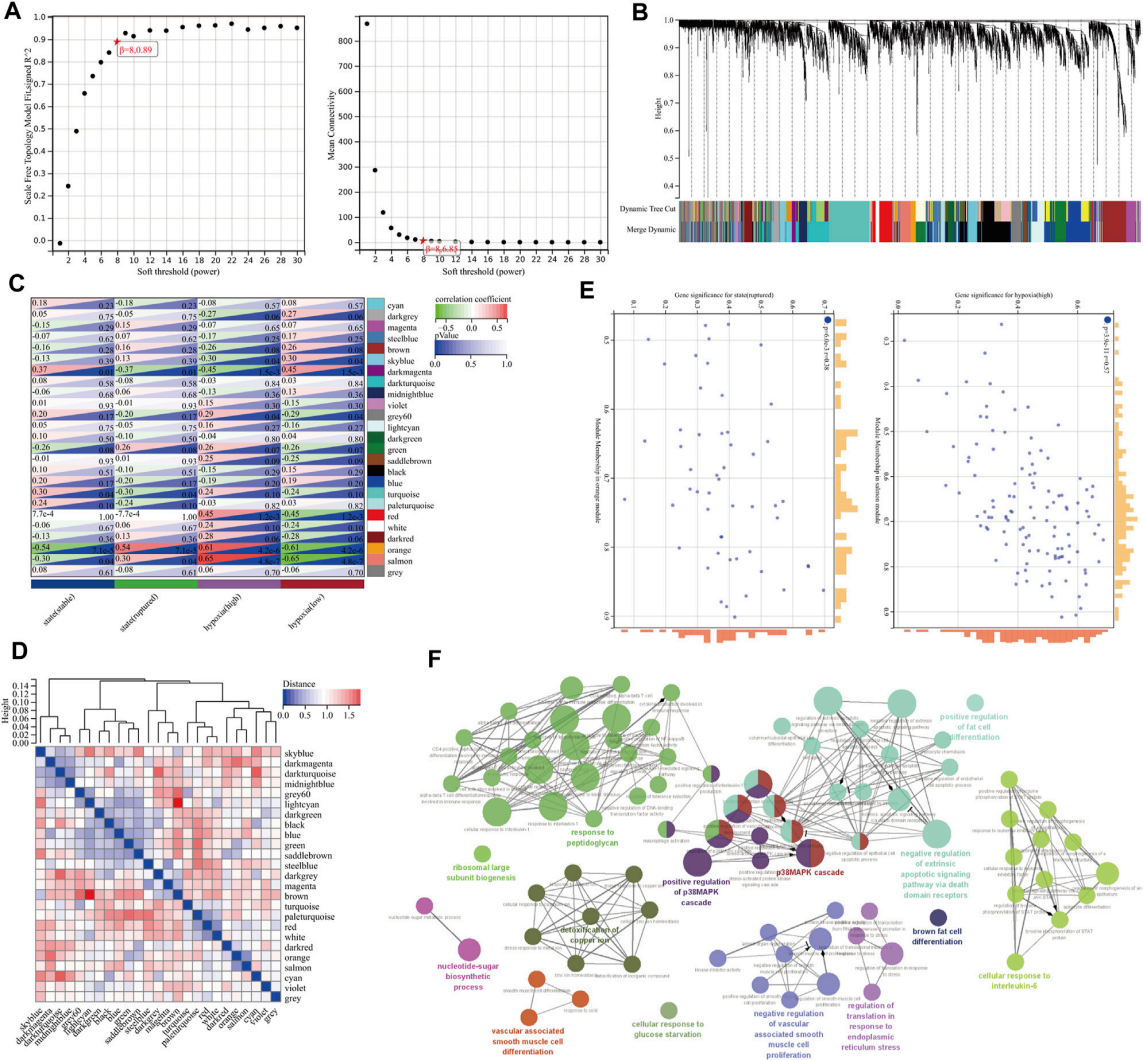
Construction of the gene coexpression network by WGCNA. **(A)** Soft-threshold power for WGCNA. The soft-thresholding power was selected from 1–20, the coefficient threshold was set to 0.89, and the soft threshold was set to 8. **(B)** Dendrogram of the gene cluster analysis of all filtered gene sets. The first row at the bottom of the dendrogram indicates the unmerged dynamic tree cut, and the second row shows the 25 merged modules, with different colors indicating different modules. **(C)** Hierarchical clustering and correlation heatmap of all module genes. The bluer the color is, the higher the correlation. **(D)** Heatmap of the correlation between different module genes and hypoxia and AAA status. Darker colors indicate higher correlations and smaller *p value*s with greater statistical significance. **(E)** A scatterplot of GS and MM scores of genes in the orange and salmon modules. **(F)** GO enrichment analysis of genes in the orange and salmon modules.

After cleaning the data in the GSE98278 dataset by WGCNA, we performed coexpression network construction analysis on 4102 genes from 48 samples. Then, the scale-free network was constructed by setting the soft threshold to 8, the independence to 0.89, and the average connectivity close to 0 (Figure 3A). A total of 25 correlated coexpression modules were obtained, where the blue module (596 genes), black module (467 genes), and dark turquoise module (401 genes) were the three most significant clusters (Figure 3B). In the 25 modules, there were good correlations in gene expression between hypoxia and the AAA signature modules, in which the salmon module had the highest correlation with hypoxia (*r* = 0.65, *p* = 4.8e-7) and had a positive correlation with rAAA (*r* = 0.30, *p* = 0.04); the orange module had the highest correlation with rAAA (*r* = 0.54, *p* = 7.1e-5) and had a high correlation with the high hypoxia group (*r* = 0.61, *p* = 4.2e-6) (Figure 3C). These correlations were confirmed by analyzing hierarchical clustering, the heatmap, and the adjacency relationships (Figure 3D).

### Identification of Hypoxia- and Ruptured Abdominal Aortic Aneurysm-Associated Hub Genes

**FIGURE 4:**
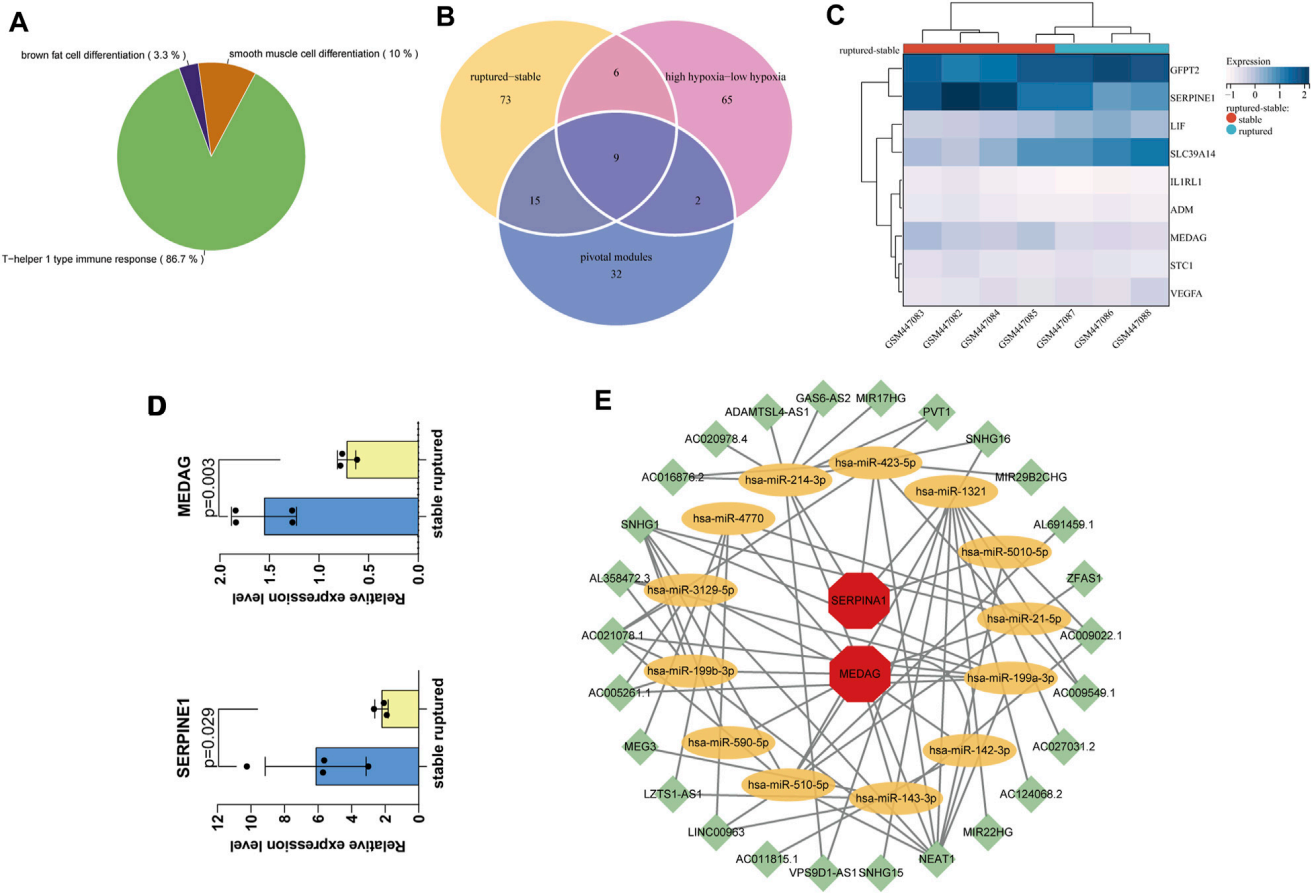
Screening and validation of hub genes. **(A)** The most relevant gene modules with coenriched GO entries of high hypoxia and rAAA. **(B)** The Venn diagram shows nine candidate genes related to hypoxia and AAA rupture in the three cohorts. **(C)** Hierarchical clustering of the nine candidate genes in the mouse dataset GSE17901, finally identifying the final hub genes MEDAG and SERPINE1. **(D)** Expression levels of MEDAG and SERPINE1 in GSE17901. **(E)** CeRNA network diagram. Green nodes indicate lncRNAs, yellow nodes indicate miRNAs, and red nodes indicate mRNAs.

The MM and GS scores showed a high positive correlation with each other in the orange module and salmon module (Figure 3E), and 58 candidate genes with a high correlation with clinical characteristics were identified from the above modules. GO functional enrichment analysis showed that the BPs could be divided into several groups, with the top three enrichment groups being regulation of translation in response to endoplasmic reticulum stress, response to peptidoglycan, and detoxification of copper ion (Figure 3F). We found that the T-helper 1 type immune response, smooth muscle cell differentiation, and brown fat cell differentiation were coenriched pathways from the orange and salmon modules (Figure 4A). The overlap of three cohort candidate genes was identified using a Venn diagram, including rAAA (Figure 4B, yellow set), high hypoxic group AAA (Figure 4B, pink set), and pivotal modules (Figure 4B, blue set). We extracted nine candidate genes (GFPT2, SERPINE1, LIF, SLC39A14, IL1RL1, ADM, MEDAG, STC1, and VEGFA) associated with rupture and high hypoxia.

### Validation of Candidate Genes

We performed differential validation of the nine candidate genes identified above using seven samples from the mouse dataset GSE17901 (Figure 4C). Among them, MEDAG (*p* = 0.003) and SERPINE1 (*p* = 0.029) were considered the final key genes, which were significantly different in the mouse dataset (Figure 4D). They were common genes appearing in both the human AAA dataset and the mouse AAA dataset. Thus, these two genes are potential biomarkers that may be involved in the molecular mechanisms of hypoxia-related AAA progression and rupture.

### Construction of the Competitive Endogenous RNA Network

We predicted miRNAs and lncRNAs that may be involved in regulating MEDAG and SERPINE1 from the starBase database (v2.0; Sun Yat-sen University, Guangzhou, China) and performed coexpression validation. Then, we obtained 58 miRNA-lncRNA and 14 miRNA–mRNA interactions. A ceRNA network containing 72 edges and 41 nodes (including 26 lncRNAs, 13 miRNAs, and two mRNAs) was constructed (Figure 4E). These miRNAs may influence the expression of MEDAG and SERPINE1 and play a role in the development of AAA.

### Prediction of Gene and Traditional Chinese Medicine Interactions

We imported the two final key genes MEDAG and SERPINE1 into the HERB database, and the gene and TCM interactions showed 93 drugs associated with the above two genes. Among these, there was only one TCM associated with MEDAG - sunflower seed; 92 medicines were associated with SERPINE1, and ginkgo seed had the smallest *p value* (Table 1).

**TABLE 1:** The TCM associated with MEDAG and SERPINE1.

Genes	Herb name	*p*-value
MEDAG	XIANG RI KUI ZI; Sunflower Seed; Helianthus annuus	0.00032
SERPINE1	BAI GUO; Ginkgo seed; Semen Ginkgo	1.18E-07
	SUO LUO ZI; Buckeye Seed; Semen Aesculi	2.88E-06
	MEI ZHOU JIN LV MEI; Virginia Witch Hazel; Hamamelis virginiana	8.00E-06
	GAO LIANG JIANG; Alpiniae Officirum Rhizome	9.84E-06
	CHA YE; Common Tea; Camellia sinensis [Syn. Thea sinensis]	1.49E-05
	MA CHI XIAN; all-grass of Purslane; Herba Portulacae	1.63E-05
	BAI BIAN DOU; White Hyacinth Bean; Semen Lablab Album	1.70E-05
	HUO MA REN; Hemp Seed; Semen Cannabis	2.83E-05
	TU FU LING; Glabrous Greenbrier Rhizome; Rhizoma Smilacis Glabrae	3.55E-05
	QIAO MAI; Common Buckwheat; Fagopyrum esculentum	3.68E-05

## Discussion

In this study, we first identified hypoxia-related candidate genes between ruptured and stable AAA by performing combination analysis with differential analysis, ssGSEA hypoxia score analysis, and WGCNA on the GSE98278 dataset, verified the differential expression of candidate genes in the GSE17901 dataset, and finally selected two hub genes, MEDAG and SERPINE1. Subsequently, the ceRNA network that regulates the expression of MEDAG and SERPINE1 was explored by predicting the miRNAs and lncRNAs that may bind to the hub genes. TCM has become increasingly popular in recent years worldwide due to its long history and multiple pharmacological effects, and there have been a large number of studies on cardiovascular diseases (Cheung, 2011; Rastogi et al., 2016). Then, we explored potential TCMs associated with MEDAG and SERPINE1 through the HERB database.

Several studies have shown that receptor–ligand interactions contribute to the development of AAA. For example, the interaction between toll-like receptor 2 (TLR2) and its endogenous ligand promotes the development of AAA (Yan et al., 2015), and the expression of RAGE and its ligand AGE is highly elevated in human aneurysm specimens (Zhang et al., 2009). In the functional enrichment analysis of the AAA ruptured and stable groups and the high and low hypoxia groups, GO showed that receptor–ligand activity was associated with rAAA and high hypoxia. KEGG analysis showed that the cytokine–cytokine receptor interaction signaling pathway was the most prominent pathway common to both. These results suggest that the interaction of hypoxia-related cytokines and their receptors may promote the development and rupture of AAA (Wang et al., 2015; Li et al., 2018).

We identified all modules and their genes in rAAA using WGCNA. The orange and salmon modules were the pivotal modules associated with AAA rupture and high hypoxia. The GO analysis of the above two modules showed that the T-helper 1-type immune response is a central pathway. In AAA, inflammatory processes play a crucial role, accompanied by cellular and humoral immunity (Prucha et al., 2019). Two T-helper cell subsets, Th1 and Th2 cells, mediate these different forms of immunity (Li et al., 2018). Previous studies have suggested that Th1 is upregulated in AAA (Prucha et al., 2019). The balance between Th1-type and Th2-type immune responses may influence the progression of many inflammatory diseases (including AAA) (Schulte et al., 2008).

It is worth mentioning that no reports have elucidated the role of MEDAG in AAA. MEDAG is a novel adipogenesis gene involved in visceral obesity that is strongly associated with the onset and progression of type 2 diabetes (Yang and Yu, 2021) and can increase the risk of obesity (D'Angelo et al., 2018), which is related to the development and rupture of AAA. Although the function of MEDAG in AAA has not been elucidated, we hypothesized that it influences AAA rupture and hypoxia by affecting lipid metabolism. This hypothesis agrees with the results of GO functional enrichment analysis of the brown fat cell differentiation pathway in our orange and salmon modules. While sunflower seed, the only TCM associated with it, has a preventive effect on acute hyperlipidemia and chronic hypercholesterolemia, it has been shown that altered lipids in the aorta lead to the development and ruptured aortic aneurysms (Saito et al., 2019). However, whether hyperlipidemia and hypercholesterolemia are associated with AAA progression is controversial (Mulorz et al., 2020; Ikezoe et al., 2021).

SERPINE1, also known as PAI-1, plays an essential protective role in AAA rupture as a significant inhibitor of tissue plasminogen activator (tPA) and urokinase-type plasminogen activator (uPA) and plays the same role in sex differences (DiMusto et al., 2012; English et al., 2015). This gene is relatively mature in AAA. Therefore, there are more TCMs associated with this gene. Ginkgo seeds may affect AAA rupture by reducing blood glucose levels through the inhibition of α-glucosidase (Sun et al., 2021). It also has anti-inflammatory, antioxidant, and ischemia/reperfusion injury treatment benefits (Pereira et al., 2013; Gargouri et al., 2018; Yan et al., 2020).

To further understand how miRNAs are involved in the regulation of MEDAG and SERPINE1 and to gain insights into these mechanisms, we constructed a ceRNA network diagram in which miR-21-5p downregulation of MEDAG and SERPINE1 was associated with AAA hypoxia and rupture, which is consistent with findings from previously published studies (Plana et al., 2020). Our study showed that SNHG1/miR-21-5p/MEDAG is a potential pathway to regulate MEDAG expression. However, further experiments are needed to demonstrate this hypothesis.

Although our study predicted that the hypoxia-related genes MEDAG and SERPINE1 are involved in the development of AAA, this study still has some limitations. First, the sample size of the dataset from the GEO database was small, with only 31 rAAA and 17 eAAA samples. Second, the validation dataset we chose was a mouse dataset, and angiotensin II-induced AAA in mice has a different inherent pathology than humans and is small in size. The screening results need to be further verified. Third, our study predicted the central genes MEDAG and SERPINE1, which are involved in the progression of AAA, with hypoxia-related genes and identified TCMs associated with the hub genes. Nevertheless, these findings need to be confirmed by *in vitro* and *in vivo* experiments in AAA.

## Conclusion

In conclusion, our study identified two hub genes (MEDAG and SERPINE1) associated with AAA hypoxia and rupture by a combination analysis of differential gene selection, ssGSEA hypoxia score, and WGCNA. We also identified a potential regulatory pathway, SNHG1/miR-21-5p/MEDAG. In addition, we predicted a potentially useful TCM association with MEDAG and SERPINE1 using the HERB database. Furthermore, our study found that pathways such as cytokine–cytokine receptor interaction and T-helper 1-type immune response are involved in AAA hypoxia and rupture.

## Data Availability

The data analyzed in the study were obtained from GEO, accession number: GSE98278 (https://www.ncbi.nlm.nih.gov/geo/query/acc.cgi?acc=GSE98278) and GSE17901 (https://www.ncbi.nlm.nih.gov/geo/query/acc.cgi?acc=GSE17901).

## Glossary

AAA: abdominal aortic aneurysm
HIF: hypoxia-inducible factor
VEGF: vascular endothelial growth factor
FLT: Fms related receptor tyrosine kinase
FLK: fetal liver kinase
ANG: angiogenin
TIE: tyrosine kinase with immunoglobulin and epidermal growth factor homology domains
WGCNA: weighted gene co-expression network analysis
GEO: gene expression omnibus
GSE: gene expression omnibus
GSEA: gene set enrichment analysis
FDP: false discovery rate
MSigDB: molecular signatures database
ssGSEA: single sample gene set enrichment analysis
GSVA: gene set variation analysis
DEGs: differential expressed genes
eAAA: elective stable abdominal aortic aneurysm
rAAA: ruptured abdominal aortic aneurysm
GO: gene ontology
KEGG: kyoto encyclopedia of genes and genomes
MAD: median absolute deviation
GS: gene significance
MM: module membership
GEPIA: gene expression profiling interactive analysis
lncRNA: long noncoding RNA
miRNA: microRNA
mRNA: messenger RNA
CeRNA: competitive endogenous RNA
TCM: traditional chinese medicine
BP: biological process
CC: cellular component
MF: molecular function
GFPT: glutamine fructose-6-phosphate amidotransferase
SERPINE: serpin family E member
LIF: leukemia inhibitory factor
SLC39A14: solute carrier family 39, member 14
IL1RL1: interleukin one receptor-like one
ADM: adriamycin-resistant
MEDAG: mesenteric estrogen-dependent adipogenesis gene
STC: stanniocalcin
VEGFA: vascular endothelial growth factor A
