# Supplementary material for: Identification of MEDAG and SERPINE1 Related to Hypoxia in Abdominal Aortic Aneurysm Based on Weighted Gene Coexpression Network Analysis
Source: Front Physiol. 2022 Jul 6;13:926508. doi: 10.3389/fphys.2022.926508 (PMC9301186; doi:10.3389/fphys.2022.926508)
Supplement: Supplementary file 1 [file Table1.DOCX]

###################

#GSE98278 dif exp & GSVA_hypoxia

###################

library("GEOquery")

options( 'download.file.method.GEOquery' = 'libcurl' )

gset <- getGEO('GSE98278',destdir = ".",AnnotGPL = T,getGPL = T)

save(gset,file = 'GSE98278.gset.Rdata')

#迅雷下载的压缩包

#读取

gset <- getGEO('GSE29676',destdir = ".",AnnotGPL = F,getGPL = F)

save(gset,file = 'GSE19860.gset.Rdata')

# 表达数据读取

dat <- read.table(file = "GSE27786_series_matrix.txt",

header =TRUE,comment.char = "!", row.names=1)

#GEO数据处理（提取表达矩阵和临床信息，样本分组）

#加载数据

load("GSE98278.gset.Rdata")

#提取临床和表达信息

pdata <- pData(gset[[1]])

table(pdata$characteristics_ch1.1)

library("stringr") #加载包，方便提取样本信息列分组

group_list <- ifelse(str_detect(pdata$characteristics_ch1.1, "ruptured"), "ruptured", "stable") #设置参考水平

group_list = factor(group_list, levels = c("stable","ruptured"))

table(group_list)

#group_list=str_split(as.character(phe$title),' ',simplify =T)[,2]

#View(group_list) #phe$title锚定title列，以空格分隔，逗号前后表示行和列

#save(raw_exprSet,group_list,file='GSE28702_raw_exprSet.Rdata')#保存工作空间

#表达

exp <- exprs(gset[[1]])

boxplot(exp,outline=FALSE, notch=T,col=group_list, las=2)

#标准化与归一化

library(limma)

exp <- neqc(exp)#做背景校正、标准化

exp=normalizeBetweenArrays(exp)

par(mar=c(8,3,4,3))

boxplot(exp,outline=FALSE, col=group_list, las=2)

range(exp)

#ID转换

index = gset[[1]]@annotation

anno <- data.table::fread("GPL10558.annot")#读取

colnames(anno)

probe2symbol <- anno[,c("ID","Gene symbol")]#取需要的列

probe2symbol$SYMBOL_ID[probe2symbol$SYMBOL_ID=='']<-NA

colnames(probe2symbol) <- c("PROBE_ID", "SYMBOL_ID")#改名，让他适合下面的自定义函数

eset <- exp %>% as.data.frame() %>% mutate(PROBE_ID=rownames(exp)) %>% select(PROBE_ID,everything())

exprSet_symbol <- merge(probe2symbol, eset, by = "PROBE_ID")

exprSet_symbol <- exprSet_symbol %>% select(-PROBE_ID) %>%

mutate(rowMean = rowMeans(.[,3:49])) %>% #去除symbol中的NA

filter(SYMBOL_ID != "NA") %>% #把表达量的平均值按从大到小排序

arrange(desc(rowMean))

# symbol留下第一个

exprSet_symbol <- exprSet_symbol %>% distinct(SYMBOL_ID,.keep_all = TRUE) %>% dplyr::select(-rowMean)

{

p2g <- function(eset,probe2symbol){

library(dplyr)

library(tibble)

library(tidyr)

eset <- as.data.frame(eset)

p2g_eset <- eset %>%

rownames_to_column(var="PROBE_ID") %>% #合并探针的信息

inner_join(probe2symbol,by="PROBE_ID") %>% #去掉多余信息

select(-PROBE_ID) %>% #重新排列

dplyr::select(SYMBOL_ID,everything()) %>% #求出平均数(这边的点号代表上一步产出的数据)

mutate(rowMean = rowMeans(.[grep("GSM", names(.))])) %>% #去除symbol中的NA

filter(SYMBOL_ID != "NA") %>% #把表达量的平均值按从大到小排序

arrange(desc(rowMean)) %>% # symbol留下第一个

distinct(SYMBOL_ID,.keep_all = TRUE) %>% #反向选择去除rowMean这一列

dplyr::select(-rowMean) %>% # 列名变成行名

column_to_rownames(var = "SYMBOL_ID")

save(p2g_eset, file = "p2g_eset.Rdata")

return(p2g_eset)

}

p2g_eset <- p2g(eset = eset, probe2symbol = probe2symbol)

load("p2g_eset.Rdata")

}

#GSVA

if (!require("BiocManager", quietly = TRUE))

install.packages("BiocManager")

BiocManager::install("GSVA")

library(GSVA)

library(GSEABase)

#读入gmt文件，这个可以从MSigDB上下载，这边选的上gene symbol根据自己的data来选择

gmt_file="./GSE98278/GSE98278_GSVA/HALLMARK_HYPOXIA.v7.5.1 (1).gmt"

geneset <- getGmt(gmt_file)

es<- gsva(expr, geneset,method= "ssgsea",

min.sz=10,max.sz=500,verbose=TRUE)

#hypoxia_DEG

library(limma)

group_hypoxia <- readClipboard() %>% as.data.frame()

colnames(group_hypoxia) <- c("group_list")

group_list = factor(group_hypoxia$group_list, levels = c("stable","ruptured"))

table(group_list)

design=model.matrix(~0+factor(group_list))

colnames(design) <- c("stable","ruptured")

fit_n=lmFit(expr,design)

cont.matrix <- makeContrasts(ruptured-stable,levels = design)

fit_n <- contrasts.fit(fit_n,cont.matrix)

fit_n <- eBayes(fit_n)

all_diff <- topTable(fit_n,number = 20000)

write.table(all_diff,file = "GSE98278_all_DEG.txt",sep = "\t",quote = F)

DEG_hypoxia <- all_diff %>% select(logFC,P.Value,adj.P.Val) %>% subset(abs(all_diff$logFC)>=1 & all_diff$P.Value<=0.05)

##########################

#PCA

##########################

library(FactoMineR)

library(factoextra)

exp <- read.table("GSE98278_DEG_EXP.txt",stringsAsFactors = F)

colnames(exp) <- exp[1,]

rownames(exp) <- exp[,1]

exp <- exp[-1,-1]

dat= as.matrix(t(exp))

dat <- apply(dat,2,as.numeric)

goup_list <- c(rep("stable",24),rep("ruptured",8),rep("stable",7),rep("ruptured",9))

group_list <- factor(goup_list,levels=c("stable","ruptured"))

dat.pca <- PCA(dat, graph = FALSE)

pca_plot <- dat.pca, geom.ind = "point", col.ind = group_list, palette = c( "#00AFBB", "#E7B800"), addEllipses = TRUE, legend.title = "Groups" )

################################

#WGCNA

################################

#if (!requireNamespace("BiocManager", quietly = TRUE))

# install.packages("BiocManager")

#BiocManager::install(c("GO.db", "preprocessCore", "impute","limma"))

#install.packages(c("matrixStats", "Hmisc", "foreach", "doParallel", "fastcluster", "dynamicTreeCut", "survival"))

#install.packages("WGCNA")

library("WGCNA") #引用WGCNA包

library("limma") #引用limma包

expFile="TCGA_rpkm.txt" #输入文件名字

normalCount=24 #正常样品数目

tumorCount=24 #肿瘤样品数目

setwd("C:\\Users\\lexb4\\Desktop\\WGCNA\\11.TCGAwgcna") #设置工作目录

#读取文件,并对输入文件整理

rt=read.table(expFile,sep="\t",header=T,check.names=F)

rt=as.matrix(rt)

rownames(rt)=rt[,1]

exp=rt[,2:ncol(rt)]

dimnames=list(rownames(exp),colnames(exp))

data=matrix(as.numeric(as.matrix(exp)),nrow=nrow(exp),dimnames=dimnames)

data=avereps(data)

data=log2(data+1)

data=data[apply(data,1,sd)>0.5,]

datExpr0=t(data)

###检查缺失值

gsg = goodSamplesGenes(datExpr0, verbose = 3)

if (!gsg$allOK)

{

# Optionally, print the gene and sample names that were removed:

if (sum(!gsg$goodGenes)>0)

printFlush(paste("Removing genes:", paste(names(datExpr0)[!gsg$goodGenes], collapse = ", ")))

if (sum(!gsg$goodSamples)>0)

printFlush(paste("Removing samples:", paste(rownames(datExpr0)[!gsg$goodSamples], collapse = ", ")))

# Remove the offending genes and samples from the data:

datExpr0 = datExpr0[gsg$goodSamples, gsg$goodGenes]

}

###样品聚类

sampleTree = hclust(dist(datExpr0), method = "average")

pdf(file = "1_sample_cluster.pdf", width = 12, height = 9)

par(cex = 0.6)

par(mar = c(0,4,2,0))

plot(sampleTree, main = "Sample clustering to detect outliers", sub="", xlab="", cex.lab = 1.5, cex.axis = 1.5, cex.main = 2)

###剪切线

abline(h = 20000, col = "red")

dev.off()

###删除剪切线以下的样品

clust = cutreeStatic(sampleTree, cutHeight = 20000, minSize = 10)

table(clust)

keepSamples = (clust==1)

datExpr0 = datExpr0[keepSamples, ]

###准备临床数据

traitData=data.frame(Normal=c(rep(1,normalCount),rep(0,tumorCount)),

Tumor=c(rep(0,normalCount),rep(1,tumorCount)))

row.names(traitData)=colnames(data)

fpkmSamples = rownames(datExpr0)

traitSamples =rownames(traitData)

sameSample=intersect(fpkmSamples,traitSamples)

datExpr0=datExpr0[sameSample,]

datTraits=traitData[sameSample,]

###样品聚类

sampleTree2 = hclust(dist(datExpr0), method = "average")

traitColors = numbers2colors(datTraits, signed = FALSE)

pdf(file="2_sample_heatmap.pdf",width=15,height=12)

plotDendroAndColors(sampleTree2, traitColors,

groupLabels = names(datTraits),

main = "Sample dendrogram and trait heatmap")

dev.off()

###power值散点图

enableWGCNAThreads() #多线程工作

powers = c(1:20) #幂指数范围1:20

sft = pickSoftThreshold(datExpr0, powerVector = powers, verbose = 5)

pdf(file="3_scale_independence.pdf",width=9,height=5)

par(mfrow = c(1,2))

cex1 = 0.9

###拟合指数与power值散点图

plot(sft$fitIndices[,1], -sign(sft$fitIndices[,3])*sft$fitIndices[,2],

xlab="Soft Threshold (power)",ylab="Scale Free Topology Model Fit,signed R^2",type="n",

main = paste("Scale independence"));

text(sft$fitIndices[,1], -sign(sft$fitIndices[,3])*sft$fitIndices[,2],

labels=powers,cex=cex1,col="red");

abline(h=0.90,col="red") #可以修改

###平均连通性与power值散点图

plot(sft$fitIndices[,1], sft$fitIndices[,5],

xlab="Soft Threshold (power)",ylab="Mean Connectivity", type="n",

main = paste("Mean connectivity"))

text(sft$fitIndices[,1], sft$fitIndices[,5], labels=powers, cex=cex1,col="red")

dev.off()

###邻接矩阵转换

sft #查看最佳power值

softPower =sft$powerEstimate #最佳power值

adjacency = adjacency(datExpr0, power = softPower)

softPower

###TOM矩阵

TOM = TOMsimilarity(adjacency);

dissTOM = 1-TOM

###基因聚类

geneTree = hclust(as.dist(dissTOM), method = "average");

pdf(file="4_gene_clustering.pdf",width=12,height=9)

plot(geneTree, xlab="", sub="", main = "Gene clustering on TOM-based dissimilarity",

labels = FALSE, hang = 0.04)

dev.off()

###动态剪切模块识别

minModuleSize = 25 #模块基因数目

dynamicMods = cutreeDynamic(dendro = geneTree, distM = dissTOM,

deepSplit = 2, pamRespectsDendro = FALSE,

minClusterSize = minModuleSize);

table(dynamicMods)

dynamicColors = labels2colors(dynamicMods)

table(dynamicColors)

pdf(file="5_Dynamic_Tree.pdf",width=8,height=6)

plotDendroAndColors(geneTree, dynamicColors, "Dynamic Tree Cut",

dendroLabels = FALSE, hang = 0.03,

addGuide = TRUE, guideHang = 0.05,

main = "Gene dendrogram and module colors")

dev.off()

###相似模块聚类

MEList = moduleEigengenes(datExpr0, colors = dynamicColors)

MEs = MEList$eigengenes

MEDiss = 1-cor(MEs);

METree = hclust(as.dist(MEDiss), method = "average")

pdf(file="6_Clustering_module.pdf",width=7,height=6)

plot(METree, main = "Clustering of module eigengenes",

xlab = "", sub = "")

MEDissThres = 0.25 #剪切高度可修改

abline(h=MEDissThres, col = "red")

dev.off()

###相似模块合并

merge = mergeCloseModules(datExpr0, dynamicColors, cutHeight = MEDissThres, verbose = 3)

mergedColors = merge$colors

mergedMEs = merge$newMEs

pdf(file="7_merged_dynamic.pdf", width = 9, height = 6)

plotDendroAndColors(geneTree, mergedColors,"Dynamic Tree Cut",

dendroLabels = FALSE, hang = 0.03,

addGuide = TRUE, guideHang = 0.05,

main = "Gene dendrogram and module colors(TCGA)")

dev.off()

moduleColors = mergedColors

table(moduleColors)

colorOrder = c("grey", standardColors(50))

moduleLabels = match(moduleColors, colorOrder)-1

MEs = mergedMEs

###模块与性状数据热图

nGenes = ncol(datExpr0)

nSamples = nrow(datExpr0)

moduleTraitCor = cor(MEs, datTraits, use = "p")

moduleTraitPvalue = corPvalueStudent(moduleTraitCor, nSamples)

pdf(file="8_Module_trait.pdf",width=6,height=6)

textMatrix = paste(signif(moduleTraitCor, 2), "\n(",

signif(moduleTraitPvalue, 1), ")", sep = "")

dim(textMatrix) = dim(moduleTraitCor)

par(mar = c(5, 10, 3, 3))

labeledHeatmap(Matrix = moduleTraitCor,

xLabels = names(datTraits),

yLabels = names(MEs),

ySymbols = names(MEs),

colorLabels = FALSE,

colors = blueWhiteRed(50),

textMatrix = textMatrix,

setStdMargins = FALSE,

cex.text = 0.5,

zlim = c(-1,1),

main = paste("Module-trait relationships(TCGA)"))

dev.off()

###计算MM和GS值

modNames = substring(names(MEs), 3)

geneModuleMembership = as.data.frame(cor(datExpr0, MEs, use = "p"))

MMPvalue = as.data.frame(corPvalueStudent(as.matrix(geneModuleMembership), nSamples))

names(geneModuleMembership) = paste("MM", modNames, sep="")

names(MMPvalue) = paste("p.MM", modNames, sep="")

traitNames=names(datTraits)

geneTraitSignificance = as.data.frame(cor(datExpr0, datTraits, use = "p"))

GSPvalue = as.data.frame(corPvalueStudent(as.matrix(geneTraitSignificance), nSamples))

names(geneTraitSignificance) = paste("GS.", traitNames, sep="")

names(GSPvalue) = paste("p.GS.", traitNames, sep="")

###批量输出性状和模块散点图

for (trait in traitNames){

traitColumn=match(trait,traitNames)

for (module in modNames){

column = match(module, modNames)

moduleGenes = moduleColors==module

if (nrow(geneModuleMembership[moduleGenes,]) > 1){

outPdf=paste("9_", trait, "_", module,".pdf",sep="")

pdf(file=outPdf,width=7,height=7)

par(mfrow = c(1,1))

verboseScatterplot(abs(geneModuleMembership[moduleGenes, column]),

abs(geneTraitSignificance[moduleGenes, traitColumn]),

xlab = paste("Module Membership in", module, "module"),

ylab = paste("Gene significance for ",trait),

main = paste("Module membership vs. gene significance\n"),

cex.main = 1.2, cex.lab = 1.2, cex.axis = 1.2, col = module)

abline(v=0.8,h=0.5,col="red")

dev.off()

}

}

}

###输出GS_MM数据

probes = colnames(datExpr0)

geneInfo0 = data.frame(probes= probes,

moduleColor = moduleColors)

for (Tra in 1:ncol(geneTraitSignificance))

{

oldNames = names(geneInfo0)

geneInfo0 = data.frame(geneInfo0, geneTraitSignificance[,Tra],

GSPvalue[, Tra])

names(geneInfo0) = c(oldNames,names(geneTraitSignificance)[Tra],

names(GSPvalue)[Tra])

}

for (mod in 1:ncol(geneModuleMembership))

{

oldNames = names(geneInfo0)

geneInfo0 = data.frame(geneInfo0, geneModuleMembership[,mod],

MMPvalue[, mod])

names(geneInfo0) = c(oldNames,names(geneModuleMembership)[mod],

names(MMPvalue)[mod])

}

geneOrder =order(geneInfo0$moduleColor)

geneInfo = geneInfo0[geneOrder, ]

write.table(geneInfo, file = "GS_MM.xls",sep="\t",row.names=F)

###输出每个模块的基因

for (mod in 1:nrow(table(moduleColors)))

{

modules = names(table(moduleColors))[mod]

probes = colnames(datExpr0)

inModule = (moduleColors == modules)

modGenes = probes[inModule]

write.table(modGenes, file =paste0("TCGA_",modules,".txt"),sep="\t",row.names=F,col.names=F,quote=F)

}

############################################

#veen

############################################

BiocManager::install("VennDiagram")

library(VennDiagram)

hub_inner=readClipboard()

deg=readClipboard()

hypoxia_deg=readClipboard()

a <- readClipboard()

b <- readClipboard()

T1<-venn.diagram(list(A=deg,B=hypoxia_deg,C=hub_inner),filename=NULL

,lwd=4,lty=1,col='white'

,fill=c('#EA86C4','#FFD06C',"#5B75B8")

,alpha= 0.8

,fontfamily = "serif"

,category = c("high hypoxia-low hypoxia ","ruptured-stable", "pivotal modules")

,cat.col="black"

,cat.fontfamily = "serif"

,cat.default.pos = "text"

,cat.pos = 0

,reverse=TRUE

)

T <- draw.pairwise.venn(8,20,2, c( "ruptured-stable","high hypoxia-low hypoxia ","pivotal modules")

,lwd=4,lty=1,col='white'

,fill=c('#ABA8DF','#E4755D',"#5B75B8")

,cat.col="black"

,cat.fontfamily = "serif"

,cat.default.pos = "outer"

,cat.pos = c(45,-45)

,scaled =FALSE)

pdf("./venn.pdf",width = 5.5,height = 5)

par(pin=c(5.5,5.5))

grid.draw(T1)

dev.off()

#组间交集元素获得

venn_list <- list(group1 = deg,group2= hypoxia_deg,group3= hub_inner)

inter <- get.venn.partitions(venn_list)

for (i in 1:nrow(inter)) inter[i,'values'] <- paste(inter[[i,'..values..']], collapse = ', ')

write.table(inter[-c(5, 6)], 'venn3_inter.txt', row.names = FALSE, sep = '\t', quote = FALSE)

####################################

#AUC

####################################

#install.packages("pROC")

library(pROC)

shiny_plotROC()

colnames(roc)[1] <- 'S'

roc <- read.csv('ROC.csv')

library(pROC)

library(tidyverse)

data(aSAH)

roc <- read.table("GSE98278/ROC-DIF/ROC.txt")

colnames(roc) <- roc[1,]

roc <- roc[-1,]

roc <- as.matrix()

roc <- as.data.frame(roc)

roc <- apply(roc,2,as.numeric)

rocobj1 <- plot.roc(roc$S, roc$LIF,

main="Statistical comparison", col="#1c61b6")

rocobj2 <- lines.roc(roc$S, roc$SLC39A14, col="#008600")

rocobj3 <- lines.roc(roc$S,roc$IL1RL1,col="red")

testobj <- roc.test(rocobj1)

text(5, 45, labels=paste("p-value =", format.pval(testobj$p.value)), adj=c(0, .8))

legend("bottomright", legend=c("PUS1", "RBM15"), col=c("#1c61b6", "#008600"), lwd=2)

legend("bottomright", legend = c("Empirical", "Binormal", "Density", "Fitdistr\n(Log-normal)"), col = c("black", "#1c61b6", "#008600", "#840000"),lwd = 2)

##########################

#GO/KEGG

##########################

BiocManager::install('clusterProfiler')

BiocManager::install("org.Hs.eg.db")

BiocManager::install("topGO")

BiocManager::install("Rgraphviz")

BiocManager::install("pathview")

library(clusterProfiler)

library(topGO)

library(Rgraphviz)

library(pathview)

library(org.Hs.eg.db)

#library(org.Mm.eg.db)

library(ggplot2)

read.table("GSE98278/GSE98278_DEG/DEG_all_list.txt",header = T,col.names = T,sep = "\t",stringsAsFactors = F)

DEG.gene_symbol <- readClipboard()

DEG.gene_symbol <- hypoxia_deg

DEG.gene_symbol = as.character(inter$Gene) #获得基因 symbol ID

DEG.entrez_id = mapIds(x = org.Hs.eg.db,

keys = DEG.gene_symbol,

keytype = "SYMBOL",

column = "ENTREZID") #需要将symbolID转换成ENTREZID

DEG.entrez_id = na.omit(DEG.entrez_id) #去掉NA值

#BP（Biological process）层面上的富集分析：

erich.go.BP = enrichGO(gene = DEG.entrez_id,

OrgDb = org.Hs.eg.db,

keyType = "ENTREZID",

ont = "BP",

pvalueCutoff = 0.05,

qvalueCutoff = 0.25)

##分析完成后，作图

dotplot(erich.go.BP)

barplot(erich.go.BP)

ggsave("3lnc-erich.go.bp")

# 树形图

plotGOgraph(erich.go.BP)

#显示GO基因集中所富集到的基因，则将该基因与GO集连线

cnetplot(enrich.go.bp, showCategory = 5)

#保存为pdf

pdf(file="./enrich.go.bp.pdf",width = 5,height = 9)

dotplot(erich.go.BP)

dev.off()

#CC分析作图

erich.go.CC = enrichGO(gene = DEG.entrez_id,

OrgDb = org.Hs.eg.db,

keyType = "ENTREZID",

ont = "CC",

pvalueCutoff = 0.05,

qvalueCutoff = 0.25)

## 画图

barplot(erich.go.CC)

##MF分析作图

erich.go.MF = enrichGO(gene = DEG.entrez_id,

OrgDb = org.Hs.eg.db,

keyType = "ENTREZID",

ont = "MF",

pvalueCutoff = 0.05,

qvalueCutoff = 0.25)

#GO分析，直接做3图拼图，一步完成

ALL <- enrichGO(gene = DEG.entrez_id,

OrgDb = org.Hs.eg.db,

keyType = "ENTREZID",

ont = "ALL",

pvalueCutoff = 0.05,

#pAdjustMethods = "BH",

qvalueCutoff = 0.25)

save(ALL,file = "DEG_GO_ALL.Rdata")

save(ALL,file = "hypoxia_DE_GO_ALL.Rdata")

save(ALL,file = "hub_GO_ALL.Rdata")

barplot(ALL,split="ONTOLOGY")+facet_grid(ONTOLOGY~.,scale="free")

pdf(file="./go.pdf",width = 6.8,height = 6.8 )

dotplot(ALL,font.size=10,split="ONTOLOGY",label_format=60)+facet_grid(ONTOLOGY~.,scale="free")

dev.off()

#去背景色和网格线

#+theme_bw()+ theme(panel.grid=element_blank())

#KEGG pathway 分析和上面介绍的GO分析是一样的只是把enrichGO()函数改成 enrichKEGG()

KEGG <- enrichKEGG(gene = DEG.entrez_id,

organism = "hsa",#mmu

keyType = "kegg",

pvalueCutoff = 0.05,

qvalueCutoff =0.25)

#字体大小，显示项数，标题，泡泡大小

dotplot(KEGG,font.size=20,showCategory=10,title="Enrichment KEGG Top10")

#字体太长解决方案

library(stringr)

barplot(ALL)+scale_x_discrete(labels=function(x) str_wrap(x,width=10))

dotplot(ALL,font.size=15, split="ONTOLOGY")+facet_grid(ONTOLOGY~.,scale="free")+scale_x_discrete(labels=function(x) stringr::str_wrap(x,width = 60))

#获得数据，自己作图

# 之前计算的结果

up <- enrichGO(gene = gs_up,

keyType = 'ENSEMBL',

OrgDb = org.Hs.eg.db,

ont = "BP",

pAdjustMethod = "BH",

pvalueCutoff = 1,

qvalueCutoff = 1,

readable = TRUE)

# 根据显著性取前10个

test=as.data.frame(up)

test=test[1:10,]

# 然后自己计算Fold Enrichment，并按照Fold Enrichment升序排序

library(stringr)

gr1 <- as.numeric(str_split(test$GeneRatio,"/",simplify = T)[,1])

gr2 <- as.numeric(str_split(test$GeneRatio,"/",simplify = T)[,2])

bg1 <- as.numeric(str_split(test$BgRatio,"/",simplify = T)[,1])

bg2 <- as.numeric(str_split(test$BgRatio,"/",simplify = T)[,2])

test$fold <- (gr1/gr2)/(bg1/bg2)

test <- arrange(test,fold)

#作图

#将description因子化方便可视化排序，原来的Description是字符型变量，作图默认按照字母顺序

test$Description = factor(test$Description,levels = test$Description,ordered = T)

ggplot(test,aes(x = fold,y = Description))+

geom_point(aes(color = p.adjust,

size = Count))+

scale_color_gradient(low = "red", high = "blue")+

xlab("Fold Enrichment")+

theme_bw()+

#edit legends

guides(

#reverse color order (higher value on top)

color = guide_colorbar(reverse = TRUE))

#reverse size order (higher diameter on top)

#size = guide_legend(reverse = TRUE))

################################

#pie plot

################################

pie_data <- c(86.67,10,3.33) %>% as.matrix(c(1,3))

colnames(pie_data) <- c("c1")

rownames(pie_data) <- c("a","b","c")

rownames(pie_data) <- c("T-helper 1 type immune response","smooth muscle cell differentiation","brown fat cell differentiation")

pie(pie_data[,1])

library(RColorBrewer)

percent <- round(pie_data[,1]/sum(pie_data[,1])*100, 1)

# 计算比例，保留一位小数

label <- paste(rownames(pie_data), "(", percent, "% )")

pie(pie_data[,1], border="white", col=brewer.pal(5, "Set3"),init.angle = 110,label=label)

par(mai=c(0.2,0.2,0.2,0.2))

pdf("./pie.pdf",width = 7.5,height = 6)

pie(pie_data[,1], border="white", col=c('#7AB561','#C67215',"#402971"),init.angle = 110,label=label)

dev.off()

#####################################

#CIBERSORT

#####################################

BiocManager::install("e1071")

BiocManager::install("preprocessCore")

setwd("./CIBERSORT/CIBESORT_Run/")

source("CIBERSORT.R")

result1 <- CIBERSORT("LM22.txt","data.txt",perm=1000,QN=T)

write.csv(result1,"./GSE98278_result1.csv")

#可视化

#

sample <- readClipboard()

state <- readClipboard()

group <- as.data.frame(sample,state)

group$group <- rownames(group)

library(pheatmap)

result2 <- result1 %>% as.data.frame() %>% mutate(state=state)

result2 <- result2 %>% subset(`P-value`<=0.05) %>% select(1:22,26)

result13 <- result2 %>% select(1:22)

pheatmap::pheatmap(result13,show_colnames = T,scale = "row",annotation_row = group)

pheatmap(exprs(gbm_es)[geneSetOrder, sampleOrderBySubtype],

show_colnames = F, cluster_cols = F,

annotation_col = pData(gbm_es[,sampleOrderBySubtype]))

#################################################################

#GSE17901

# Version info: R 3.2.3, Biobase 2.30.0, GEOquery 2.40.0, limma 3.26.8

################################################################

# Differential expression analysis with limma

library(GEOquery)

library(limma)

library(umap)

# load series and platform data from GEO

gset <- getGEO("GSE17901", GSEMatrix =TRUE, AnnotGPL=TRUE)

save(gset,file = 'GSE17901.gset.Rdata')

if (length(gset) > 1) idx <- grep("GPL4134", attr(gset, "names")) else idx <- 1

gset <- gset[[idx]]

# make proper column names to match toptable

fvarLabels(gset) <- make.names(fvarLabels(gset))

# group membership for all samples

gsms <- "XXXXXXXXXXXXXXXXX0000111XXXXXXXXXXX"

sml <- strsplit(gsms, split="")[[1]]

# filter out excluded samples (marked as "X")

sel <- which(sml != "X")

sml <- sml[sel]

gset <- gset[ ,sel]

# log2 transformation

ex <- exprs(gset)

qx <- as.numeric(quantile(ex, c(0., 0.25, 0.5, 0.75, 0.99, 1.0), na.rm=T))

LogC <- (qx[5] > 100) ||

(qx[6]-qx[1] > 50 && qx[2] > 0)

if (LogC) { ex[which(ex <= 0)] <- NaN

exprs(gset) <- log2(ex) }

ex <- exprs(gset) <- normalizeBetweenArrays(exprs(gset)) # normalize data

#phe

ExperimentData <-gset@experimentData

rm(ExperimentData)

phe <-gset@phenoData@data

#ID

library(tidyverse)

featureData<- gset@featureData@data

featureData<-select(featureData,1:3)

expr <- expr %>% inner_join()

ex <- as.data.frame(ex)

ex$ID <- rownames(ex)

probe2symbol <- featureData[,c("ID","Gene.symbol")]#取需要的列

colnames(probe2symbol) <- c("ID", "SYMBOL_ID")#改名，让他适合下面的自定义函数

expr_normal <- merge(probe2symbol,ex,by = "ID")

expr_normal <- expr_normal %>% as.matrix()

rownames(expr_normal) <- expr_normal[,"SYMBOL_ID"]

expr_normal <- expr_normal[,-c(1:2)]

rownames <- rownames(expr_normal)

expr <- expr_normal %>% apply(2,as.numeric)

rownames(expr) <- rownames(expr_normal)

#去重

expr <- avereps(expr)

expr <- as.data.frame(expr) %>% data.matrix() %>% as.data.frame()

expr_log <- log2(expr+1)

write.table(expr,file = "./GSE17901/GSE17901_expr.txt")

# assign samples to groups and set up design matrix

gs <- factor(sml)

groups <- make.names(c("stable","ruptured"))

levels(gs) <- groups

gset$group <- gs

design <- model.matrix(~group + 0, gset)

colnames(design) <- levels(gs)

fit <- lmFit(gset, design) # fit linear model

# set up contrasts of interest and recalculate model coefficients

cts <- paste(groups[1], groups[2], sep="-")

cont.matrix <- makeContrasts(contrasts=cts, levels=design)

fit2 <- contrasts.fit(fit, cont.matrix)

# compute statistics and table of top significant genes

fit2 <- eBayes(fit2, 0.01)

tT <- topTable(fit2, adjust="fdr", sort.by="B", number=250)

tT <- subset(tT, select=c("ID","adj.P.Val","P.Value","t","B","logFC","Gene.symbol","Gene.title"))

write.table(tT, file=stdout(), row.names=F, sep="\t")

# Visualize and quality control test results.

# Build histogram of P-values for all genes. Normal test

# assumption is that most genes are not differentially expressed.

tT2 <- topTable(fit2, adjust="fdr", sort.by="B", number=Inf)

hist(tT2$adj.P.Val, col = "grey", border = "white", xlab = "P-adj",

ylab = "Number of genes", main = "P-adj value distribution")

# summarize test results as "up", "down" or "not expressed"

dT <- decideTests(fit2, adjust.method="fdr", p.value=0.05)

# Venn diagram of results

vennDiagram(dT, circle.col=palette())

# create Q-Q plot for t-statistic

t.good <- which(!is.na(fit2$F)) # filter out bad probes

qqt(fit2$t[t.good], fit2$df.total[t.good], main="Moderated t statistic")

# volcano plot (log P-value vs log fold change)

colnames(fit2) # list contrast names

ct <- 1 # choose contrast of interest

volcanoplot(fit2, coef=ct, main=colnames(fit2)[ct], pch=20,

highlight=length(which(dT[,ct]!=0)), names=rep('+', nrow(fit2)))

# MD plot (log fold change vs mean log expression)

# highlight statistically significant (p-adj < 0.05) probes

plotMD(fit2, column=ct, status=dT[,ct], legend=F, pch=20, cex=1)

abline(h=0)

################################################################

# General expression data analysis

ex <- exprs(gset)

# box-and-whisker plot

ord <- order(gs) # order samples by group

palette(c("#1B9E77", "#7570B3", "#E7298A", "#E6AB02", "#D95F02",

"#66A61E", "#A6761D", "#B32424", "#B324B3", "#666666"))

par(mar=c(7,4,2,1))

title <- paste ("GSE17901", "/", annotation(gset), sep ="")

boxplot(ex[,ord], boxwex=0.6, notch=T, main=title, outline=FALSE, las=2, col=gs[ord])

legend("topleft", groups, fill=palette(), bty="n")

# expression value distribution

par(mar=c(4,4,2,1))

title <- paste ("GSE17901", "/", annotation(gset), " value distribution", sep ="")

plotDensities(ex, group=gs, main=title, legend ="topright")

# UMAP plot (dimensionality reduction)

ex <- na.omit(ex) # eliminate rows with NAs

ex <- ex[!duplicated(ex), ] # remove duplicates

ump <- umap(t(ex), n_neighbors = 3, random_state = 123)

par(mar=c(3,3,2,6), xpd=TRUE)

plot(ump$layout, main="UMAP plot, nbrs=3", xlab="", ylab="", col=gs, pch=20, cex=1.5)

legend("topright", inset=c(-0.15,0), legend=levels(gs), pch=20,

col=1:nlevels(gs), title="Group", pt.cex=1.5)

library("maptools") # point labels without overlaps

pointLabel(ump$layout, labels = rownames(ump$layout), method="SANN", cex=0.6)

# mean-variance trend, helps to see if precision weights are needed

plotSA(fit2, main="Mean variance trend, GSE17901")
